# RNA structure in alternative splicing regulation: from mechanism to therapy: RNA structure-mediated splicing regulation and therapy

**DOI:** 10.3724/abbs.2024119

**Published:** 2024-07-22

**Authors:** Nengcheng Bao, Zhechao Wang, Jiayan Fu, Haiyang Dong, Yongfeng Jin

**Affiliations:** MOE Laboratory of Biosystems Homeostasis & Protection and Innovation Center for Cell Signaling Network College of Life Sciences Zhejiang University Hangzhou 310058 China

**Keywords:** aberrant splicing, antisense oligonucleotides (ASOs), RNA secondary structure, RNA-targeted therapy, splicing regulation

## Abstract

Alternative splicing is a highly intricate process that plays a crucial role in post-transcriptional regulation and significantly expands the functional proteome of a limited number of coding genes in eukaryotes. Its regulation is multifactorial, with RNA structure exerting a significant impact. Aberrant RNA conformations lead to dysregulation of splicing patterns, which directly affects the manifestation of disease symptoms. In this review, the molecular mechanisms of RNA secondary structure-mediated splicing regulation are summarized, with a focus on the complex interplay between aberrant RNA conformations and disease phenotypes resulted from splicing defects. This study also explores additional factors that reshape structural conformations, enriching our understanding of the mechanistic network underlying structure-mediated splicing regulation. In addition, an emphasis has been placed on the clinical role of targeting aberrant splicing corrections in human diseases. The principal mechanisms of action behind this phenomenon are described, followed by a discussion of prospective development strategies and pertinent challenges.

## Introduction

The remarkable biological complexity of eukaryotes is largely attributable to the intricate regulatory networks that dynamically control protein isoform expression in a spatio-temporal manner. As an integral part of gene expression, alternative splicing is a form of post-transcriptional regulation capable of generating diverse mature RNA transcripts and functional protein isoforms from a single gene. Alternative splicing was first reported for the mRNA of adenovirus 2 and was soon proven to be a prevalent mechanism across eukaryotes [1]. Moreover, organismal complexity showed a strong positive correlation with the proportion of genes undergoing alternative splicing [2]. Species with greater tissue and cellular heterogeneity tend to exhibit higher levels of alternative splicing events on a genome-wide scale. Notably, approximately 95% of human genes are estimated to undergo alternative splicing. Furthermore, since a substantial proportion of alternative splicing events are tissue specific, the resulting splicing isoforms often exhibit subtle or radical differences in functional attributes [3]. Consequently, alternative splicing plays an indispensable role in cellular differentiation and organismal development by increasing the functional diversity of the transcriptome and proteome.

The regulation of pre-mRNA splicing is a complex multistep process. This process entails precise excision of introns and the joining of exons, both of which are orchestrated by a highly complex macromolecular assembly known as the spliceosome. Previous extensive reviews have elucidated the mechanisms underlying spliceosome assembly and pre-mRNA splicing [ 4‒ 6]. In brief, the spliceosome recognizes and binds to the exon-intron junctions (5′-splice site and 3′-splice site) of nascent pre-mRNAs, as well as to intronic branch points and polypyrimidine tracts. This facilitates exon recognition and executes two successive transesterification reactions, resulting in intron removal and exon ligation. The core of the process is the definition of the 5′- and 3′-splice sites of the exon. Based on differences in exon definitions, alternative splicing events can be categorized into the following five types: exon skipping, intron retention, alternative 5′ or 3′ splice site usage, and mutually exclusive splicing [1]. Remarkably, human introns range in length from several to hundreds of kilobases, with an average size of ~5 kb, and they are interspersed with numerous ‘decoy’ splice sites that show significant consensus sequence similarity with authentic splice sites. These ‘decoy’ splice sites have the potential to induce the inclusion of pseudo-exons [ 7, 8]. Despite the prevalence of decoys, splicing proceeds with extraordinary fidelity, and it is strongly argued that additional sequence features surrounding core splicing signals contribute to exon-intron definition [9].

In addition to canonical splice site signals, alternative splicing is also regulated by splicing regulatory elements (SREs) in pre-mRNAs. On the one hand, SREs can be recognized by sequence-specific RNA-binding proteins (RBPs), which then recruit core splicing factors either by direct interaction [10] or by establishing exclusion zones [11] to influence exon definition and spliceosome assembly. On the other hand, the interactions between SREs and RBPs can be altered by the complex secondary and tertiary structure of RNA. It can affect alternative splicing by modulating RNA-ligand or RNA-protein interactions and by forming various preferred conformations [12]. Recent discoveries in biochemistry, genomics and bioinformatics are gradually revealing the critical regulatory functions of RNA structure in splicing control at the transcriptional level [ 13‒ 15]. At the same time, an increasing number of reports indicate a strong correlation between the loss of functional RNA structure and various human diseases [ 16‒ 18]. The RNA secondary structure has emerged as another critical regulatory layer governing splicing control.

This review primarily elaborates on the molecular mechanisms by which RNA secondary structure affects gene splicing modes and unveils the causal relationship between aberrant RNA conformations and disease phenotypes due to splicing defects. This paper also discusses the non-canonical regulatory mechanisms that influence secondary structure formation and splicing regulation. Given the widespread existence of splicing defects in diseases, therapeutic approaches targeting aberrant RNA splicing have been explored, with a particular focus on the potential of antisense oligonucleotides (ASOs). This review summarizes the mechanisms underlying this strategy and highlights the current clinical advancements made in combating aberrant splicing-associated diseases. In essence, this review endeavors to deepen the reader’s understanding of the molecular mechanisms underlying RNA secondary structure-mediated regulation of splicing while providing insight into the potential and clinical viability of RNA therapies designed based on this principle.

## The Roles of RNA Structure in Alternative Splicing

### Blocking/exposure of splicing signals

**Figure 1:**
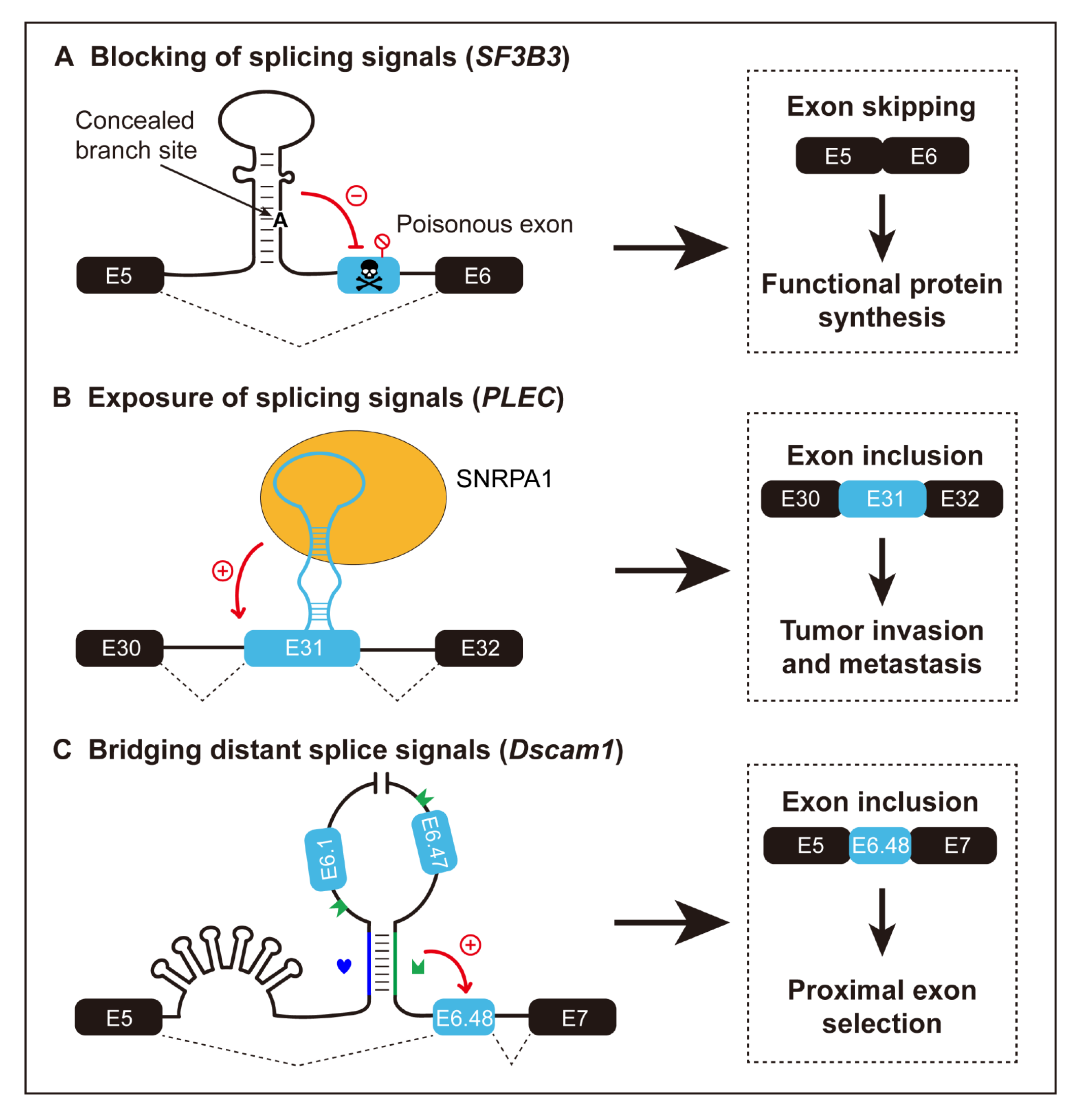
Figure 1 The RNA structure-mediated regulation of splicing signals (A) Structured RNA masking branch site signal to prevent poisonous exon inclusion. (B) Structured RNA serves as a splicing enhancer, recruiting RBP-SNRPA1 to drive a prometastatic alternative splicing program in cancer. (C) Long-range RNA secondary structures mediating spatial approximation to facilitate splicing decisions in Dscam1. Docking site (blue heart) and selector sequence (green crown).

Typically, splicing signals tend to be more effective in single-stranded environments than in double-stranded regions [19]. Genome-wide analyses have revealed numerous highly conserved RNA secondary structures that overlap with exon-intron junctions [20]. Additionally, regions around splice sites exhibit an elevated GC content [21], which can further promote the formation of stable structures capable of masking splice signals. A well-studied example is the microtubule-associated protein Tau (MAPT), which utilizes a hairpin structure to interfere with the recognition of the 5′ splice site in exon 10, thereby maintaining balanced expression of the 3R-tau and 4R-tau isoforms [22]. Perturbations that disrupt the balance ratio have been implicated in driving tauopathy pathogenesis [23]. In addition, sequestering the stem structure of the exon 7 splice site in *SMN2* indirectly contributes to the onset of spinal muscular atrophy (SMA). This structure negatively affects the recruitment of U1 snRNP to the 5′ splice site in exon 7, partially explaining why *SMN2* is unable to produce sufficient functional products to compensate for the loss of *SMN1* in SMA patients [24]. This RNA structure-based strategy for splice site modulation could also be extended to other splicing signals. A study by Tse *et al*. [25] revealed a three-way junction structure located at the 3′ end of intron 15 in the factor VIII ( *F8*) gene. This higher-order structural element sequesters polypyrimidine tracts and thus attenuates the recognition of the 3′ splice site signal. As a consequence, the proper splicing of exon 16 in *F8* results in increased vulnerability to mutations that could lead to hemophilia A. Moreover, hiding the branch site within the stem loop has been proposed as a common mechanism for preventing the aberrant inclusion of poisonous exons, with particular attention given to *SF3B3*, ensuring its physiological function in DNA repair [26] ( Figure 1A).

Although splicing signals in an unstructured environment may expose them more readily, this does not necessarily favor their recognition. Unstructured regions may make splicing signals vulnerable to disruptive factors [19]. To mitigate such detrimental effects, RNA structures can be employed to constrain and stabilize the single-stranded conformation around splicing signals, thereby enhancing their recognition. SREs downstream of stable hairpin structures tend to exhibit elevated activity regardless of their sequence identity [27]. In addition, many RNA stem-loop structures have been shown to facilitate recognition of the loop sequence by being exposed in a single-stranded conformation. For example, the highly structured EDA exon of *fibronectin* can form a stem-loop that presents an exonic splicing enhancer (ESE) via a single-stranded loop to facilitate binding by the splicing factor SRSF1 [28]. The binding of several positive regulatory factors (B52, SRp55, and NOVA-1) [ 29‒ 31] and negative regulatory factors (hnRNP A1) [32] involved in splicing has also been shown to be dependent on the RNA secondary structure and the target nucleotide sequences. This structural presentation effect largely accounts for the splicing defects caused by some mutations, although these mutations do not alter SREs [33]. RNA secondary structures themselves can also serve as splicing enhancers, interacting with RNA-binding proteins and participating in splicing regulation. Enriched RNA structural elements on exon 31 of *PLEC* interact with the RBP SNRPA1 to enhance exon inclusion. This leads to the expression of rod domain-containing plectin isoforms, which can increase cancer cell invasion and promote metastasis [34] ( Figure 1B).

Pre-mRNAs with specific secondary structures can mask or expose splicing signals in different ways, thereby regulating the accessibility of RBPs to SREs. However, it is worth noting that the relationship between RNA secondary structures and RBPs is inherently bidirectional. RBPs can reciprocally influence the conformations of prefolded RNA secondary structures, engendering dynamic structural rearrangements that affect the outcome of alternative splicing. A paradigmatic illustration of this intricate interplay is the alternative splicing of exon 7 in the *MALT1* gene. Stem-loop structures flanking the exon 7 splice sites are subject to competitive binding by the RBPs hnRNP U and hnRNP L, which exert opposing effects on structural stability. hnRNP U binding stabilizes a conformation that precludes exon 7 recognition, whereas hnRNP L disrupts this structure, enabling the recruitment of U2AF65 and consequent exon inclusion [35]. This example highlights the dynamic and bidirectional crosstalk between preformed RNA structures and splicing regulatory factors in controlling alternative splicing decisions, increasing the complexity of the regulatory networks that govern splicing.

### Bridging distant splice signals

Previous discussions have focused mainly on locally occurring base pairing. However, the average length of human introns spans thousands of nucleotides and sometimes reaches hundreds of thousands of bases [36]. This vast sequence space underscores the critical capacity of long-range RNA structures to coordinate splicing regulation across distal regions. Complex RNA structures can enable the spatial approximation of distant segments along one RNA transcript by base pairing across thousands of nucleotides. It bridges two spatially separate splice signals, the 5′ splice site and the 3′ splice site, bringing them in close proximity to facilitate requisite splicing decisions [37].

In this situation, the RNA secondary structure does not involve reorganization of proteins but serves only to bridge upstream and downstream splice signals to facilitate their recognition. A prime illustration of this mechanism is provided by Down syndrome cell adhesion molecule 1 ( *Dscam1*) in *Drosophila*, where extensive long-range RNA pairings coordinate the alternative splicing of its four exon clusters (containing 12, 48, 33, and 2 variable exons) through mutually exclusive splicing, potentially generating more than 38,000 distinct mRNA isoforms [38]. As illustrated in the exon 6 clusters ( Figure 1C), the conserved docking site is located downstream of constitutive exon 5, whereas discrete selector sequences reside adjacent to each variable exon 6. Through base pairing between the docking site and a given selector sequence, the flanking splice site of the chosen exon 6 is positioned in spatial proximity, thereby promoting its preferential inclusion in the maturing mRNA transcript [ 39‒ 41]. Such RNA-mediated juxtaposition of splice sites clarifies the basis for the stochastic selection of a single exon 6, despite the compatibility of the incorporation of multiple variants that are compatible with each mRNA isoform. Similarly, a comparable structural code was deciphered in the exon 4 clusters of *Dscam1* [42]. However, in the exon 9 clusters, only a few scattered structures have been validated [ 43, 44], while the splicing regulatory mechanisms of most variable exons remain largely unelucidated and need to be further explored and elucidated. Remarkably, recent studies have revealed another set of hidden RNA secondary structures within exon 6 clusters [45]. These structures cooperate pairwise with docking site-selector sequences to form a multidomain pre-mRNA conformation that favors the inclusion of distal exon 6 while impeding the pairing of proximal exon 6, thereby suppressing its inclusion. This mechanism ingeniously counteracts the “first-come, first-served” splicing principle and facilitates a balanced stochastic choice of *Dscam1* splice variants. Last, long-distance RNA-RNA interactions have also been demonstrated in genes such as *14-3-3ξ* [42], *MHC* [42], *SF1* [46], *DNM1* [47], *PLP1* [48], *ATE1* [49], and others to have an activating effect of bringing splicing signal elements closer together.

### Looping out the exons

**Figure 2:**
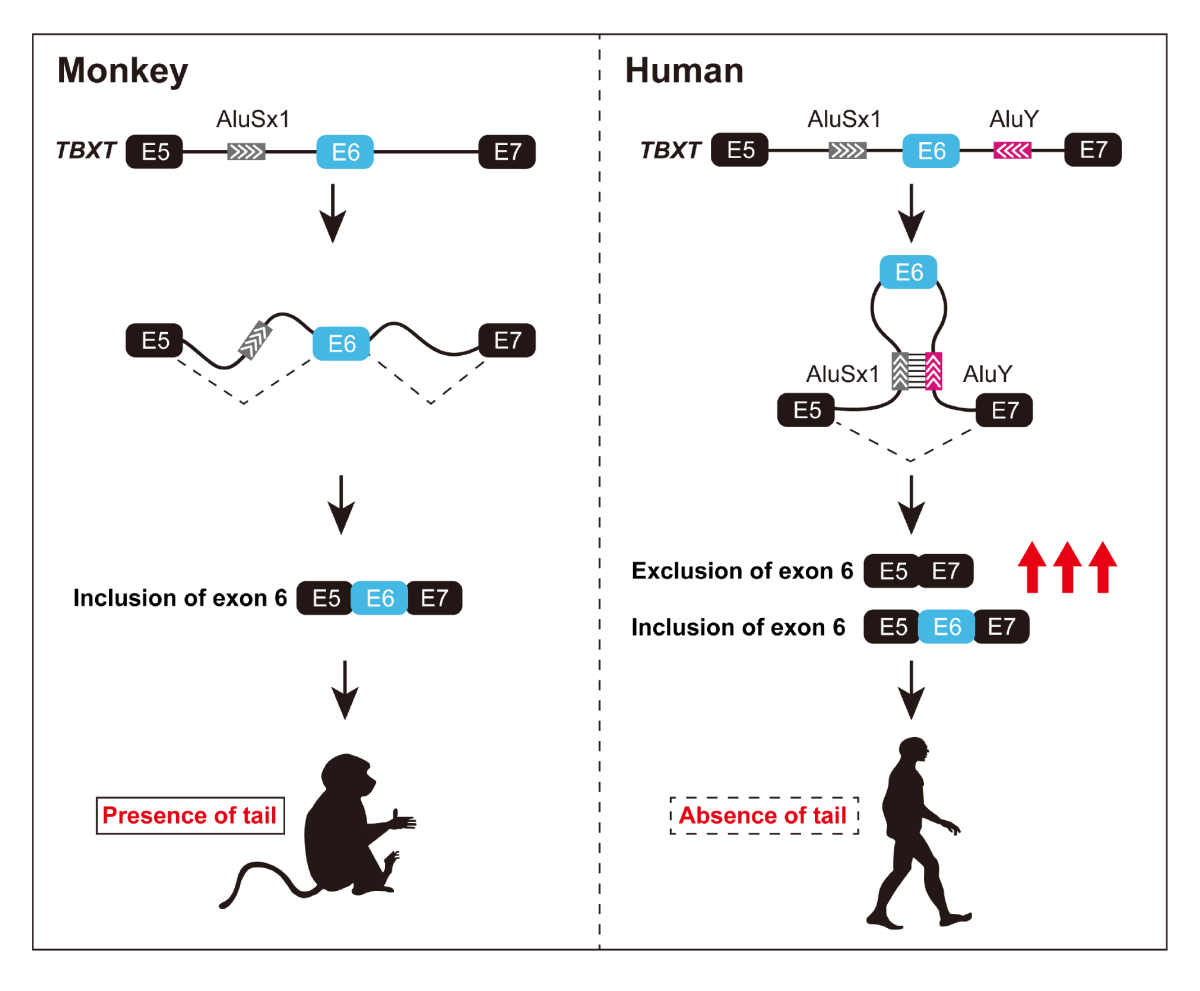
Figure 2 The genetic mechanism of tail loss evolution in hominoids via *Alu*-mediated exon looping-out The upper panels depict the normal splicing pattern in monkeys, where exons 5, 6, and 7 are properly included in the mature mRNA transcript (left), and the alternative looping-out event in hominoids caused by the insertion of an Alu element, resulting in the skipping of exon 6 (right). The lower panels represent the consequences of the alternative splicing event, where the inclusion of exon 6 generates a full-length functional protein allowing tail development in nonhominoid primates (left), while its exclusion in hominoids results in a truncated, nonfunctional protein leading to tail loss during evolution (right).

If the stem-loop formed by the RNA secondary structure encompasses the entire exon, it can induce exon skipping by looping out of the exon by flanking intron base pairing. The most prevalent and illustrative example of this mechanism is the regulatory role of intronic *Alu* elements in alternative splicing. As primate-specific retrotransposons, *Alu* elements are more enriched in intronic regions flanking variable exons than in those flanking constitutive exons [50]. These intronic *Alus* elements can form long-distance RNA duplexes that bring the intervening splice sites in close proximity, thereby looping out and suppressing the expression of the internal exons. In addition, this process is always accompanied by the formation of back-spliced (circularized) exons, which significantly increases the complexity of circular RNAs [ 51 , 52]. Returning to the main point, such a transformation may confer new functions on the gene and play a regulatory role at certain times or in specific cell types. For instance, a recent study revealed a specific *AluY* element located downstream of exon 6 in the human *TBXT* gene, which is uniquely present in all hominids (humans and apes) but absent in other primates [53]. This *AluY* element can engage in reverse complementary base pairing with an ancient *AluSx1* element located upstream of the exon to form a stable stem-loop structure. This interaction gives rise to the hominoid specific, in-frame splicing isoform *TBXT ^Δexon6^*. The exon-skipped transcript is sufficient to induce a tail-deficient phenotype. To a certain extent, this research sheds light on the molecular genetic basis underlying the evolutionary loss of tails in humans and apes [53] ( Figure 2). In addition to *Alu* element-mediated complementary pairing-induced exon skipping, a similar regulatory mode has been identified for other genes ( *DNM1* [47] , *FGFR1* [54], *Dscam1* [39], and *14-3-3ζ* [42]), and it appears to be even more evolutionarily conserved.

### Repeat-associated RNA structure

Short tandem repeats (STRs), also known as microsatellite repeats, are frequent entities in many transcripts. These repeats are primarily composed of 2‒10 base pairs and are highly polymorphic [55]. Within genomic DNA, STRs tend to form unusual quadruplex-like, slipped-strand structures and imperfect hairpins. However, the presence of these noncanonical structures promotes DNA replication infidelity and substandard DNA repair. This leads to expansions of the STRs [ 56, 57]. In some cases, pathological events are induced when the number of repeats exceeds a critical threshold quantity [58].

**Figure 3:**
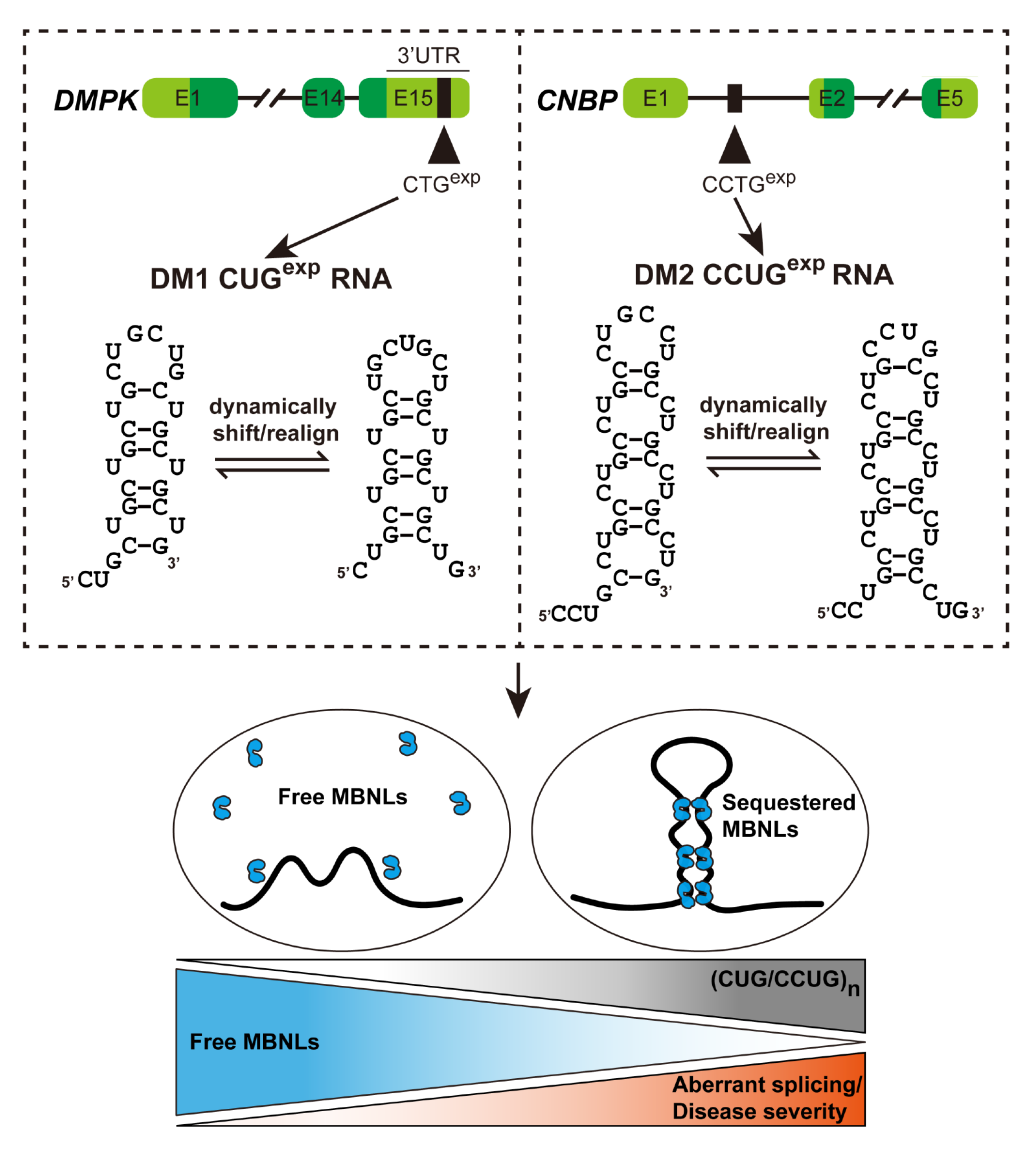
Figure 3 Models of DM1 and DM2 disease mechanisms The expanded CTG/CCTG repeats in the DMPK and CNBP genes can be transcribed into toxic RNAs, which can dynamically shift and realign “slippery hairpin” structures. The splicing regulator MBNL is sequestered by these RNAs in ribonuclear aggregates known as foci. As the number of CUG/CCUG repeats increases, the sequestration of MBNL leads to a gradual decrease in the concentration of free MBNL protein, resulting in enhanced mis-splicing and ultimately leading to the symptoms of myotonic dystrophy.

To date, pathogenic expansions of STRs have been implicated in more than 30 heritable neurodegenerative disorders [23]. The expressions of these noncoding microsatellite expansion RNAs (RNA ^exp^) can promote neuronal deterioration through various pathogenic mechanisms. Typically, transcripts containing extensively amplified repeats are prone to aberrantly folding into persistent ribonucleoprotein aggregates [59]. Coalescence of such unstable structures results in the formation of cytotoxic nuclear inclusions, sequestering and segregating vital splicing regulators that mediate the expression of modulated splicing isoforms. This leads to a global dysregulation of RNA metabolism, with the resultant aberrant splicing being implicated in a variety of patient symptoms [18]. Myotonic dystrophy type 1 (DM1) and type 2 (DM2) are disorders triggered by aberrant splicing due to the presence of repeat-associated RNA structures. Patients exhibit muscle weakness with prolonged contractions, as well as a variety of neurological symptoms, including intellectual disability, dementia, and neurofibrillary tangles [60]. The underlying cause of DM1 lies in the abnormal expansion of CTG repeats within the 3′ UTR of the *DMPK* gene, whereas DM2 arises from the expansion of CCTG repeats present in intron 1 of the *CNBP* [ 61, 62] ( Figure 3). Transcription of these repeats leads to the synthesis of C(C)UG ^exp^ RNAs, which are capable of forming metastable stem-loop structures. With the accumulation of these tandem repeats, the stability of these metastable stem-loop structures is further enhanced. These “slippery hairpins” act as sequestering sites for the muscleblind-like (MBNL) family of RBPs [18]. The retention of these MBNLs leads to their functional loss, which results in splicing defects in downstream transcripts, such as *CLCN1* and *INSR* [63]. This leads to the expression of improperly spliced isoforms and the manifestation of DM symptoms [ 62, 64‒ 66]. This RNA structure-mediated dysfunction, which was originally proposed and experimentally corroborated in the context of myotonic dystrophy, has now become a central paradigm for understanding the pathogenesis of these repeating expansion disorders [18]. Notably, spinocerebellar ataxias (SCAs) comprise more than 40 classified subtypes, 13 of which are attributed to the expansion of diverse microsatellite repeats in coding and non-coding regions across separate gene loci [67]. In SCA10, the expansion of a pentanucleotide ATTCT repeat in intron 9 of the *ATXN10* gene is associated with this gene. Studies have demonstrated the colocalization of AUUCU-expanded RNA with hnRNP K [68]. The sequestration of hnRNP K by pyrimidine-rich AUUCU-expanded RNA results in its functional impairment and may contribute to cerebellar degeneration in SCA10 pathology [69]. Moreover, the expansion of the GGCCTG hexanucleotide repeat within intron 1 of the *NOP56* gene in SCA36 patients can also sequester SRSF2 [70]. Similar pathogenic mechanisms have been proposed for other repeat expansion disorders, such as Fuchs’ endothelial corneal dystrophy [ 71, 72], Huntington’s disease [ 73‒ 75], and C9orf72-mediated amyotrophic lateral sclerosis/frontotemporal dementia (C9-ALS/FTD) [ 76‒ 78].

### *Trans*-acting RNA-RNA base pairing

We have discussed how certain RNA structures within a single transcript affect its splicing. However, is it possible that *trans*-acting RNA-RNA base pairing between different transcripts could influence alternative splicing patterns? Currently, small RNAs, such as small nucleolar RNAs (snoRNAs), microRNAs (miRNAs), and small interfering RNAs (siRNAs), are believed to play a pivotal role in this process.

**Figure 4:**
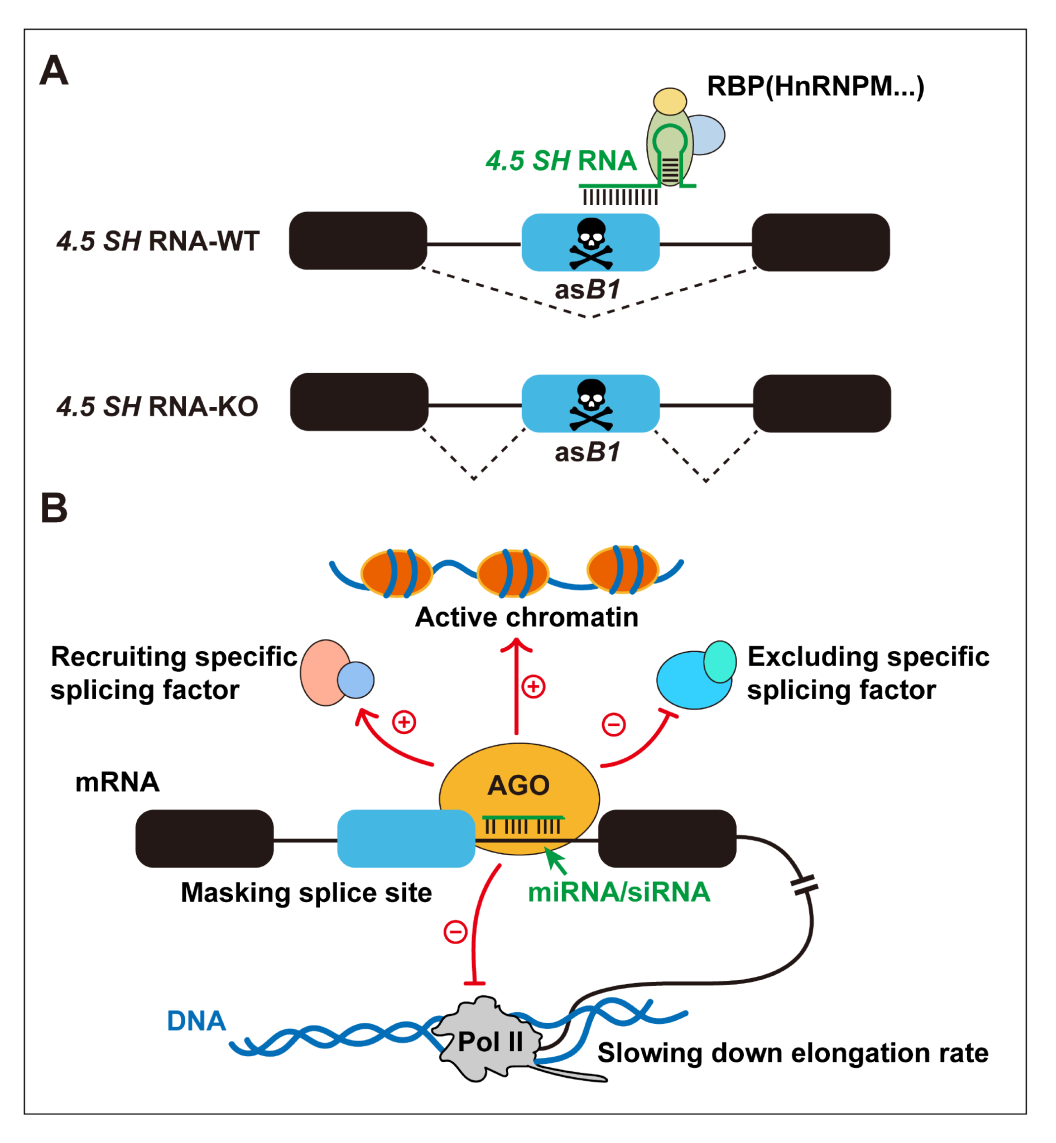
Figure 4 Regulation of alternative splicing by small RNAs through *trans*-acting RNA-RNA interactions (A) Illustration depicting the trans-acting mechanism mediated by snoRNA-4.5 SH RNA in rodents. The 4.5SH RNA recognizes and binds to poisonous exons generated by asB1. It can recruit effector proteins, including HnRNPM, to prevent deleterious exonization of asB1 in mice. (B) The role of miRNAs/siRNAs in influencing pre-mRNA splicing decisions. Guided by the trans-acting interaction of miRNA/siRNA, AGO binds to mRNA target sequences, potentially modulating splicing by masking splice sites and recruiting/excluding specific splicing factors. Alternatively, it may indirectly influence splicing patterns by slowing the elongation rate of Pol II or altering the chromatin status of transcriptionally active genes.

snoRNAs are an ancient class of 60- to 300-nucleotide-long noncoding RNAs that are known for their involvement in ribosomal RNA maturation [ 79, 80]. In mammals, while a small subset of snoRNAs involved in rRNA processing are transcribed independently from their own promoters, the majority of snoRNAs are expressed from introns of host genes [ 81, 82 ]. Recent research has revealed the regulatory role of snoRNAs in splicing through a *trans*-acting RNA-RNA base pairing mechanism [ 83– 86]. Computational analysis of large-scale human RNA-RNA interaction datasets suggested that approximately 30% of detected snoRNAs interact with their host transcripts. Notably, a substantial proportion of snoRNA-host duplexes are situated in close proximity to alternatively spliced exons and exhibit notable sequence conservation, implying that they may be involved in the regulation of splicing processes [87]. Furthermore, snoRNAs can regulate the mRNA splicing of genes located on different chromosomes. For instance, *HBII-52* snoRNA partially blocks a silencer located in its complementary region within exon Vb, thereby promoting the utilization of exon Vb to generate a functional isoform of serotonin receptor 2C. Conversely, as observed in Prader-Willi syndrome patients, the absence of *HBII-52* leads to the utilization of the proximal splice site within exon V. This results in a frameshift mutation that generates a truncated receptor lacking the critical carboxyl-terminal region essential for G protein coupling, leading to a significant reduction (10- to 100-fold) in its affinity for G proteins [84]. This phenomenon contributes to the elucidation of the molecular pathogenesis of Prader-Willi syndrome. Interestingly, a special phenomenon has been observed in a group of small, short-lived rodents, including mice and rats. These animals possess a unique snoRNA- *4*. *5SH* RNA that recognizes and pairs with poisonous exons generated by the antisense insertions of the retrotransposon *SINE B1* (as *B1*). By suppressing the use of the 3′ splice site, snoRNAs prevent the inclusion of poisonous exons during splicing ( Figure 4A). This important *trans*-acting mechanism is considered to be a self-healing repair system in rodents that protects against potentially harmful genomic insertions [88].

miRNAs or siRNAs are renowned for their involvement in RNAi, a mechanism that guides Argonaute (AGO) proteins (which constitute the catalytic core of the RNA-induced silencing complex, RISC) to target RNAs through highly flexible base pairing, thereby inducing RNA-induced gene silencing [89]. This process plays a significant role in posttranscriptional gene regulation. However, recent research has revealed an additional role for miRNAs/siRNAs in influencing pre-mRNA splicing decisions [90]. In addition to modulating the expression of specific splicing isoforms [ 91‒ 93] and splicing factors [ 94‒ 96] through RNA-induced gene silencing, AGO proteins interact with a plethora of RBPs, many of which are involved in the splicing process [90]. Consequently, the guidance of AGO proteins by small RNAs may have a deeper impact on splicing, primarily associated with mechanisms that potentially influence splice site selection [ 97, 98], the recruitment of splicing factors [ 90, 99], a decrease in the RNA polymerase (Pol) II elongation rate [ 90, 100, 101 ], and changes in the chromatin status of transcriptionally active genes [ 100, 102] ( Figure 4B).

## Beyond Sequence: Additional Factors that Reshape RNA Structure

### RNA modification

Chemical modifications have been well studied in structural RNAs such as rRNA and tRNA, and these modifications are evolutionarily conserved [ 103, 104]. As our understanding of RNA modifications has grown over the years, it has become increasingly clear that chemical modifications pervade all domains of life and mark a wide range of diverse RNA species, including tRNAs, rRNAs, mRNAs, srRNAs, snoRNAs, snRNAs, and lncRNAs. Recent advancements in characterizing the internal chemical modifications of eukaryotic mRNAs have revealed their influence on pre-mRNA splicing, adding a novel layer of regulation to alternative splicing [105].

**Figure 5:**
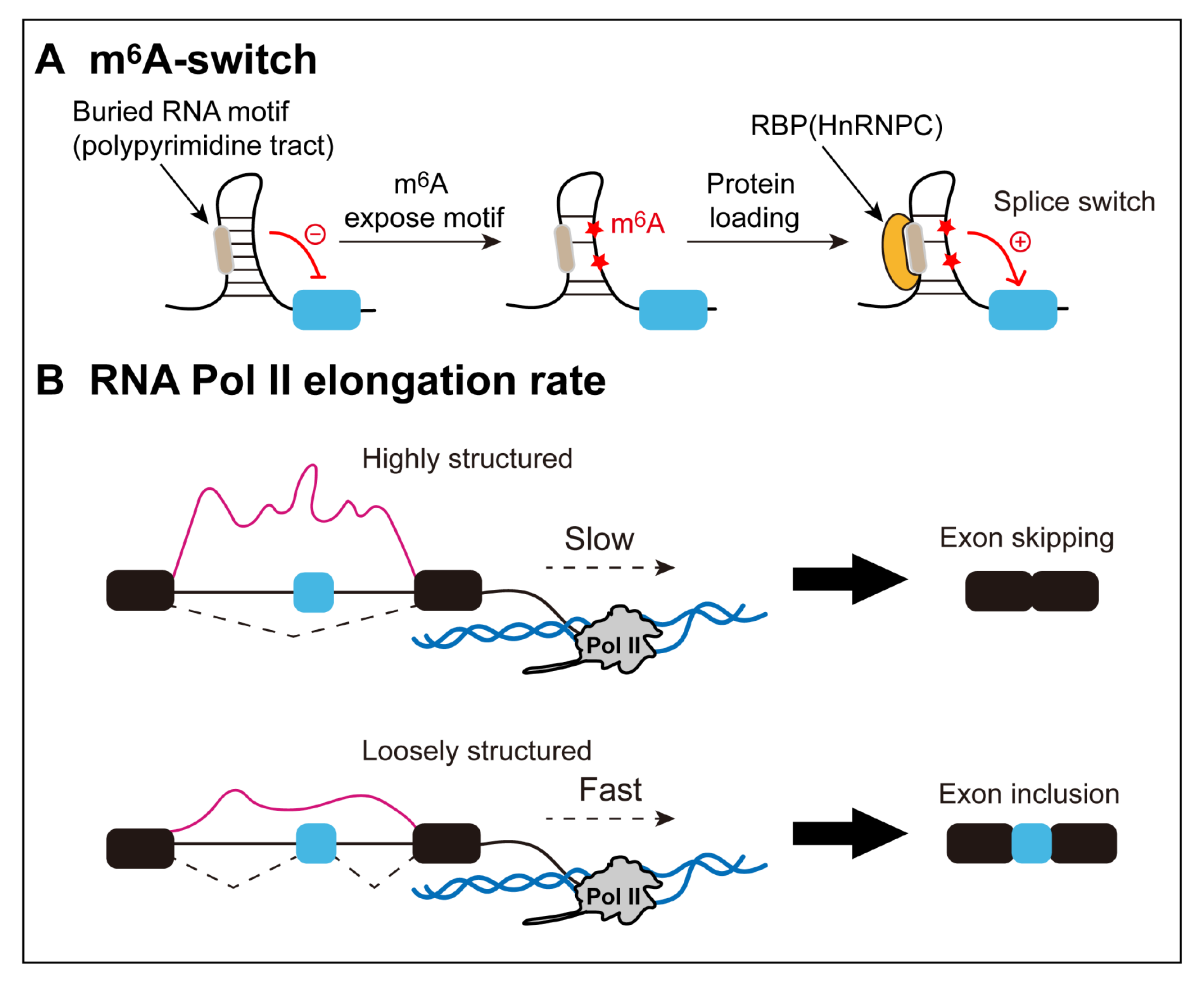
Figure 5 Additional factors that reshape RNA structure (A) RNA secondary structure potentially masks RNA motifs but can be disrupted by m6A modification to facilitate RBP recognition and binding for splicing regulation. (B) Transcription dynamics affect pre-mRNA splicing by regulating RNA structural conformation.

To date, 13 different chemical modifications of mRNA transcripts have been described, but the most widespread and abundant modification is N ^6^-methyladenosine (m ^6^A) [106]. m ^6^A modification can affect virtually all stages of the mRNA life cycle, including processing and maturation, translation, and decay and degradation [ 107‒ 111]. m ^6^A-modified mRNAs can be decoded by m ^6^A readers, most of which reside in the cytoplasm, with the exception of nuclear YTHDC1 [112]. Early studies have implicated YTHDC1-m ^6^A in alternative splicing and mRNA export, with numerous m ^6^A-mediated splicing regulatory processes being discovered [ 113‒ 116]. YTHDC1 can facilitate exon inclusion in target mRNAs by recruiting the pre-mRNA splicing factor SRSF3 while impeding the binding of SRSF10 to the mRNA [ 108, 117]. Notably, the requirement of m ^6^A and its reader YTHDC1 for female-specific alternative splicing of the sex lethal ( *Sxl*) gene determines sex bias in *Drosophila* [114]. Moreover, the presence of m ^6^A induces alterations in the secondary structure of RNA molecules that regulate the binding affinity and recruitment of RNA-binding proteins to pre-mRNAs. As described previously, RBPs typically modulate alternative splicing by binding to single-stranded SREs. However, binding may be sterically occluded if the SRE is sequestered within local RNA secondary structures. m ^6^A modifications have been demonstrated to thermodynamically destabilize RNA duplex structures [118], inducing structural remodeling and modulating the accessibility of SREs. Some have defined the mechanism that regulates the interactions between SREs and RBPs via m ^6^A-mediated RNA structural remodeling as the “m ^6^A switch” [119] ( Figure 5A). Currently, the hnRNP family of proteins involved in this process is best characterized as hnRNPC, hnRNPG, and hnRNPA2B1 [ 119‒ 121]. Liu *et al* . [122] identified the m ^6^A site within the hairpin stem of the human lncRNA *MALAT1* (metastasis-associated lung adenocarcinoma transcript 1). Through RNA structural probing assays and base mutation experiments, they demonstrated that the m ^6^A residue disrupts the stability of the *MALAT1* hairpin-stem, rendering the opposing polypyrimidine tracts single-stranded and accessible to hnRNPC. Furthermore, researchers have investigated the combined impact of knocking down (KD) METTL3/14 (the RNA methyltransferases) and hnRNPC on splicing, discovering that exons affected by the combined loss of the two proteins are more frequently located near m ^6^A switches [119]. This finding suggested that m ^6^A may function as an RNA structural remodeler that influences the splicing and maturation of mRNAs by disrupting the binding activities of posttranscriptional regulators.

In addition to m ^6^A, other modifications, such as pseudouridine (ψ) and methylation of the ribose hydroxyl group (Nm), can influence the structure of the spliceosomal snRNA and its interaction with the pre-mRNA, thus affecting the binding of spliceosomal proteins [ 123, 124]. Overall, these studies underscore the importance of RNA chemical modifications in modulating splicing through structural elements. Although many modification marks also appear in regions predicted not to form structures, future RNA structure maps will provide deeper insights into the proportion of modified bases at structured versus unstructured sites [120].

### RNA Pol II elongation rate

Another issue to keep in mind when considering the structure of pre-mRNAs is that splicing can occur cotranscriptionally [ 125, 126]. This means that slowly folding RNA structures (even if quite stable) may not have sufficient time to form in the pre-mRNA before splicing occurs. Therefore, the rate of transcription may affect pre-mRNA processing by influencing RNA structural arrangements.

The elongation rate of Pol II has been shown to significantly influence alternative splicing. Slow Pol II mutation can enhance splicing, whereas rapid Pol II mutation can reduce constitutive splicing [127]. A negative correlation between elongation rate and splicing efficiency has been consistently confirmed [ 127‒ 129]. Researchers have proposed a “window of opportunity” model to elucidate the effect of elongation rate on splicing [130]. Specifically, slower elongation expands the “window of opportunity” for recognizing weak splice sites, thereby increasing the inclusion of exons, whereas faster elongation weakens the competitive advantage of upstream exons and facilitates exon skipping. However, Fong and colleagues reported that slow and fast Pol II mutations have similar impacts on alternative exon inclusion or skipping in human cells, challenging the explanatory power of the “window of opportunity” model [131]. They concluded that an optimal transcriptional elongation rate is required to ensure the accuracy and fidelity of the splicing process. This needs to be elaborated with the involvement of additional regulatory factors.

As first proposed by Eperon *et al*. [132], the rate of RNA synthesis can influence the formation of its secondary structure. Generally, slow Pol II elongation favors sequential folding, whereas fast elongation allows nonsequential pathways to yield larger loops. Changing the elongation rate will affect both the transient and final structures adopted [ 133‒ 136]. Given that the RNA secondary structure can exert regulatory effects on splicing, transcription rates can also have a significant impact on splicing ( Figure 5B). A typical example is the complex secondary structure system in the human *ATE1* gene. This system involves five conserved regulatory intronic elements, R1–R5 [49]. Among them, R1 and R4 compete with R3 for base pairing, mediating mutually exclusive splicing of exons 7a and 7b. Moreover, R2 and R5 form an ultralong-range RNA structure spanning 30 kb to control isoform bias. Notably, slowing the Pol II elongation rate allows sufficient time for RNA folding to facilitate R2-R5 interactions, thereby increasing the exon 7a/7b ratio. However, when R2 and R5 are in close proximity to each other in a minigene construct at a distance of ~2 kb, slowing the elongation rate has no significant effect on the exon 7a/7b isoform ratio. Therefore, the elongation rate can alter the structural conformations adopted by the nascent transcript by modulating the window of opportunity for upstream sequences to base pair with complementary downstream elements, especially for long-range secondary structures. Overall, as transient RNA structures undergo multiple rounds of unpairing and reformation prior to maturation, suboptimal transcription kinetics may lead to improper folding, disrupting the expected secondary structure [137]. This can lead to uncontrolled exposure or masking of SREs, which can alter splicing patterns and yield functionally inactivated transcripts.

There is a new emerging perspective that Pol II kinetics can indirectly reshape RNA secondary structure by modulating m ^6^A abundance. This could regulate alternative splicing modes, although the mechanism has not yet been formally demonstrated [105]. This notion stems from the idea that suboptimal transcription rates elevate m ^6^A levels in transcripts [138], which may affect the recognition of SREs or the formation of specific structural elements. This finding offers an additional mechanism for how Pol II dynamics may modulate splicing. Slow transcription also enhances A-to-I editing, a process that targets double-stranded RNA structures [139]. This editing may have profound consequences for alternative splicing outcomes, especially for highly structured *Alu* elements. These changes make cryptic splice sites within *Alu* elements more similar to canonical sites, leading to the exonization of *Alu* elements [140].

Finally, from the perspective of RNA structure alone, the multifaceted effects of elongation rate on splicing are likely to involve a highly complex interplay that encompasses not only RNA folding thermodynamics but also dynamic transcript modifications and potentially other regulatory layers. The continued emergence of such additional variables beyond the “window of opportunity” or “first-come, first-served” models emphasizes that splicing modulation reflects an integrative, multiparametric response to transcription kinetics rather than a simple race between splicing and elongating polymerases.

## Base Pairing for Therapeutic Interventions in Aberrant Splicing

**Figure 6:**
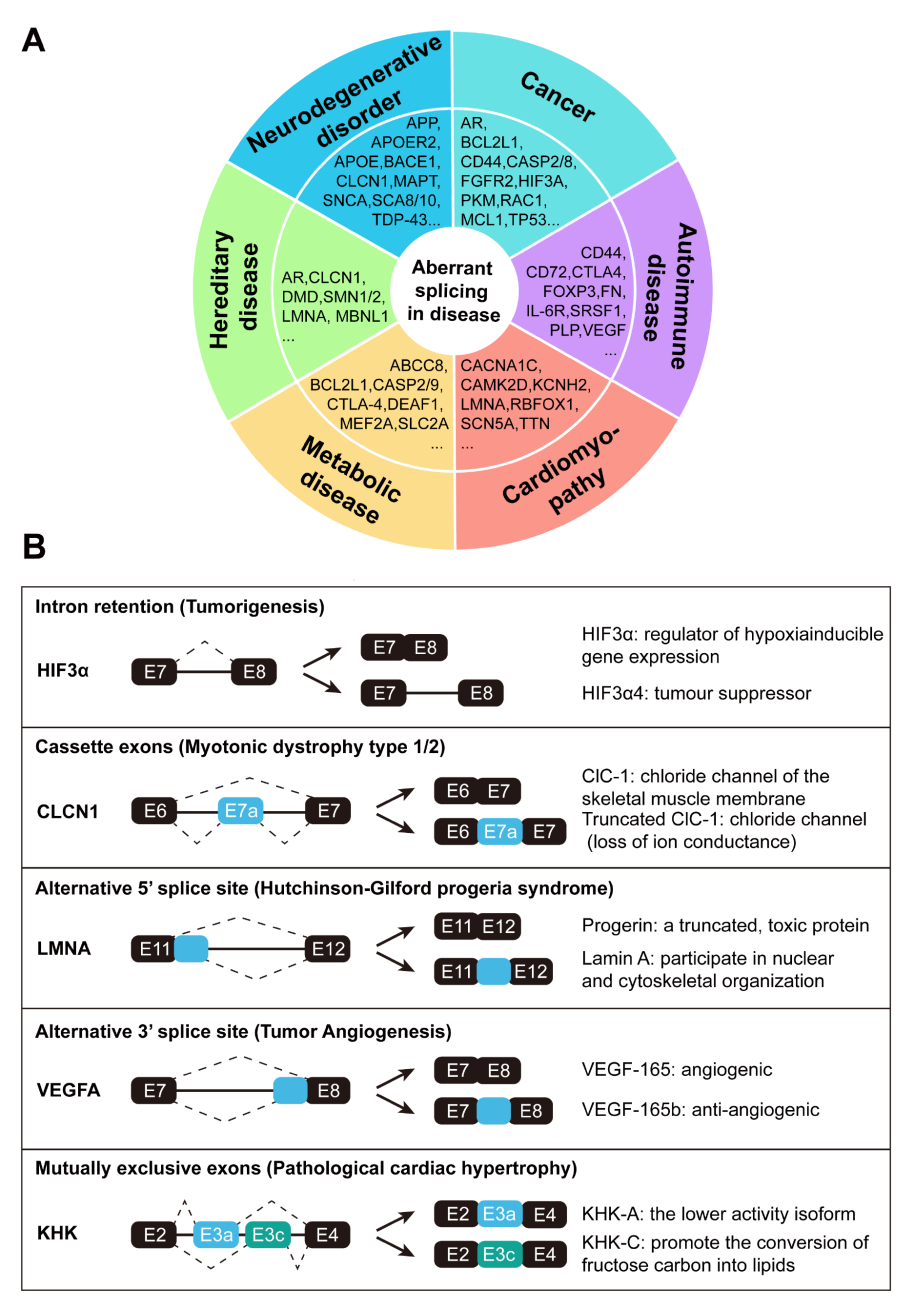
Figure 6 Splicing aberrations and pathogenesis (A) Representative genes in six major disease categories due to aberrant splicing. (B) Classes of alternative mRNA-processing events and alternatively spliced products with distinct functions in disease progression.

Aberrant splicing is prevalent and has been identified as a prominent hallmark of numerous human diseases, including the dysregulation of cholesterol homeostasis, systemic sclerosis, impaired cardiac development, cancer, and diverse neurological or muscular disorders [ 141‒ 144 ] ( Figure 6A). Different splicing variants often play distinct roles in physiological functions, thereby affecting the onset and progression of diseases ( Figure 6B). Given that splicing defects exist widely and are highly relevant to pathogenesis, the development of therapies targeting aberrant RNA splicing has become imperative. To this end, efforts are underway to explore small molecules and other approaches to advance splicing-targeting therapeutics. One promising strategy involves utilizing small nucleic acids that base-pair with the target pre-mRNA to correct aberrant splicing. For instance, Yoshimoto *et al*. [88] recently developed a novel method using *4* . *5SH* chimeric RNA as a programmable splicing regulatory factor to induce target exon skipping to rescue aberrant splicing. Furthermore, the most widely applied approach in this field is ASOs, which target the sequence of transcripts by base pairing to hinder the formation of RNA structural regulatory elements or the interaction between splicing regulatory proteins and RNA, thus achieving the goal of splicing regulation. This approach has become the focus of splice-targeting therapeutic strategies [ 145, 146].

**Figure 7:**
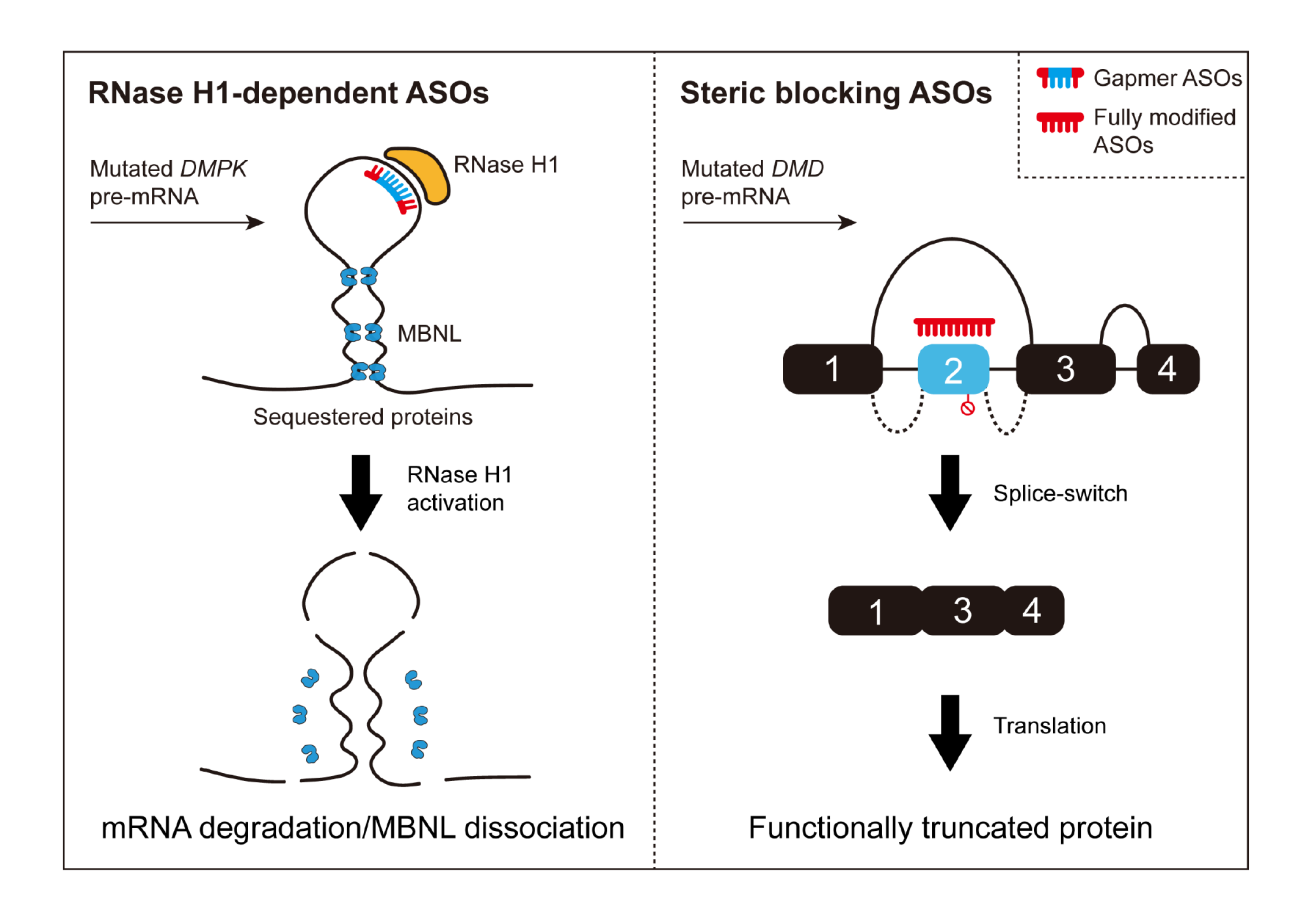
Figure 7 Molecular therapeutic mechanisms of ASOs in RNA therapy Based on their mechanisms of action, ASOs can be divided into two classes: RNase H1-dependent ASOs (left) and steric blocking ASOs (right). On the left, the abnormally mutated DMPK transcript contains an excessive number of CUG repeat sequences, which form “slippery” hairpins with high affinity for the MBNL protein, leading to its sequestration and inactivation. By employing RNase H1-dependent ASOs specifically designed to degrade these aberrant transcripts, the sequestered MBNL proteins can be liberated, thus restoring their functional role in regulating transcript processing. On the right, in patients with DMD, out-of-frame mutations result in the absence of dystrophin protein expression, leading to severe DMD phenotypes. Steric-blocking ASOs can strategically target specific exon splice site, inducing exon skipping. This approach restores the downstream open reading frame, allowing the translation of a novel, corrected, truncated mRNA that permits the production of a partially truncated yet functional dystrophin protein.

ASOs are short (~12‒25 nucleotides), single-stranded synthetic nucleic acid polymers designed to hybridize specifically with target RNA transcripts [146]. Based on their mechanisms of action, ASOs can be divided into two main classes: RNase H1-dependent ASOs and steric blocking ASOs [ 147, 148 ]. RNase H1-dependent ASOs, referred to as chimeric or gapmer ASOs, typically form RNA-DNA heteroduplexes with target RNA sequences and contain chemically modified nucleotides in flanking regions capable of being recognized and cleaved by RNase H1, leading to the degradation of the target RNA and subsequent gene silencing. In contrast, steric blocking ASOs are designed with fully modified base sequences that cannot trigger RNase H1 recognition and cleavage. Instead, they can regulate alternative splicing patterns of the target mRNA through base pairing that masks splice sites or regulatory elements [149] ( Figure 7). Therefore, steric blocking ASOs, also known as splice-switching oligonucleotides (SSOs), are being actively explored for therapeutic splicing modulation in diseases associated with RNA splicing defects, particularly in cancer and neurological or muscular disorders [150].

The first application of oligonucleotides for splicing modulation in humans occurred in 2009; these oligonucleotides target Duchenne muscular dystrophy (DMD), a severe degenerative genetic disease affecting muscles [151]. Patients were administered intramuscular injections of a 2′-OMe/PS-modified 18-mer AON (PRO051, later known as drisapersen), designed to skip exon 51 of the dystrophin gene, with the aim of restoring truncated functional protein expression. Unfortunately, it ultimately failed to meet its primary clinical endpoints and was terminated in a late-stage (phase III) clinical trial [152]. However, in 2016, another company, Sarepta Therapeutics, developed an AON with the same splicing-modulating mechanism of action, called eteplirsen, which uses a PMO (morpholino) backbone for preventing the degradation of the nuclease by cellular RNases. This AON was successfully approved for use in Duchenne patients by the U.S. Food and Drug Administration (FDA), making it the world’s first approved splicing-modulating oligonucleotide drug [153]. This pioneering strategy not only offers new hope for DMD patients but also paves the way for modulating the splicing patterns of other disease-related genes. Similarly, in tauopathies, ASOs have been developed to modulate *MAPT* expression levels in the brain, with the potential to target alternative splicing and correct imbalances in the 3R-tau/4R-tau ratio [154].

In the context of cancer treatment, the reprogramming of energy metabolism is considered a hallmark of cancer [155]. Notably, pyruvate kinase (the rate-limiting enzyme in the glycolytic pathway) undergoes splicing conversion (from *PKM1* to *PKM2*) in many tumor tissues [156]. PKM2 promotes aerobic glycolysis to support cancer cell growth and survival. In an effort to reverse this oncogenic splicing switch, researchers gained insight into the regulatory mechanisms regulating *PKM* splicing. They developed ASOs targeting exon 10 splicing enhancers contained in *PKM2*, which successfully induced a switch in splicing from *PKM2* to *PKM1* at the cellular level, promoting apoptosis in cancer cells [ 157, 158].

Furthermore, in an orthotopic hepatocellular carcinoma xenograft mouse model, the induction of *PKM* splice switching via ASOs successfully inhibited tumorigenesis without observable toxicity [159]. Similarly, Dou *et al*. [160] attempted to modulate *Bcl-x* splicing for therapeutic benefit. They utilized SSOs binding upstream of the 5′ splice site in *Bcl-x* exon 2 to increase the proapoptotic Bcl-xS/antiapoptotic Bcl-xL ratio. This ratio is crucial because its dysregulation is associated with chemoresistance and adverse outcomes across a wide range of cancers [161]. This approach effectively increased the level of the proapoptotic protein Bcl-xS while compromising the level of the antiapoptotic protein Bcl-xL. The induction of Bcl-xS by SSOs triggered apoptosis and autophagy in glioblastoma cells.

To date, the U.S. FDA has approved nine ASO drugs to be marketed for the treatment of a variety of diseases, including DMD (eteplirsen [153], golodirsen [162], viltolarsen [163], and casimersen [164]), SMA (nusinersen [165]), hereditary transthyretin amyloidosis (inotersen [166]), ALS (tofersen [167]), cytomegalovirus retinitis (fomivirsen [168]), and homozygous familial hypercholesterolemia (mipomersen [169]). Of these, five are SSOs (eteplirsen, golodirsen, casimersen, viltolarsen and nusinersen). The remaining four ASOs are RNase H-dependent (fomivirsen, mipomersen, inotersen and tofersen). Apart from several approved SSO drugs, researchers continue to develop novel SSOs with promising therapeutic potential, some of which have progressed to clinical trials ( Table 1).

**Table:** **Table 1** Summary of SSOs that have been clinically investigated and/or ASOs approved by the FDA for market entry

Disease	Drug/leadcandidate	Molecular target	ASO mechanism	Development stage	Ref.
DMD	Eteplirsen	*DMD*	Splicing modulation via steric hindrance (exon 51 skipping)	Approved by FDA in 2016	[153]
Golodirsen	*DMD*	Splicing modulation via steric hindrance (exon 53 skipping)	Approved by FDA in 2019	[162]
Viltolarsen	*DMD*	Splicing modulation via steric hindrance (exon 53 skipping)	Approved by FDA in 2020	[163]
Casimersen	*DMD*	Splicing modulation via steric hindrance (exon 45 skipping)	Approved by FDA in 2021	[164]
SMA	Nusinersen	*SMN2*	Splicing modulation via steric hindrance (exon 7 inclusion)	Approved by FDA in 2016	[165]
Hereditary transthyretin amyloidosis	Inotersen	*TTR*	mRNA degradation mediated by RNase H	Approved by FDA in 2018	[166]
ALS	Tofersen	*SOD1*	mRNA degradation mediated by RNase H	Approved by FDA in 2023	[167]
Cytomegalovirus retinitis	Fomivirsen	*HCMV Major IE transcriptional unit*	mRNA degradation mediated by RNase H	Approved by FDA in 1998	[168]
Homozygous familial hypercholesterolemia	Mipomersen	*apoB*- *100*	mRNA degradation mediated by RNase H	Approved by FDA in 2013	[169]
Batten disease	Milasen	*CLN7*	Splicing modulation via steric hindrance (Pseudoexon skipping)	“N-of-1” clinical trial	[170]
Ataxia telangiectasia	Atipeksen (AT-008)	*ATM*	Splicing modulation via steric hindrance (Pseudoexon skipping)	“N-of-1” clinical trial	[ 171, 172]
DMD	Kyndrisa	*DMD*	Splicing modulation via steric hindrance (Exon 51 skipping)	Phase III	[ 152, 173– 175]
DMD	Vesleteplirsen (SRP-5051)	*DMD*	Splicing modulation via steric hindrance (Exon 51 skipping)	Phase II	[176]
Leber’s congenital amaurosis 10	Sepofarsen (QR-110)	*CEP290*	Splicing modulation via steric hindrance (Pseudoexon skipping)	Phase II/III	[ 177, 178]
Usher syndrome	Ultevursen (QR-421a)	*USH2A*	Splicing modulation via steric hindrance (Exon 13 skipping)	Phase II/III	[179]
Dravet syndrome	Zorevunersen (STK-001)	*SCN1A*	Splicing modulation via steric hindrance (Exon 20 skipping)	Phase I/IIA	[ 180, 181]
Cystic fibrosis	SPL84	*CFTR*	Splicing modulation via steric hindrance (Pseudoexon skipping)	Phase I	[182]

ASO-based therapy has achieved significant success in RNA therapeutics by utilizing the precision of ASOs to target specific RNA sequences without causing permanent changes to the genome. This high specificity, due to their customizable sequence, ensures precise targeting and minimizes off-target effects [183]. Additionally, ASOs show great potential for treating rare diseases and emergent viral infections due to their quick design, low toxicity, and cost-effective production [184]. These qualities make ASO-based therapies highly adaptable and potent for a broad range of genetic and viral diseases, thereby advancing targeted treatment and personalized medicine. However, advances in ASO therapies continue to encounter numerous challenges. One significant challenge lies in the delivery of oligonucleotide-based therapies, particularly in the central nervous system. ASOs encounter considerable difficulty in traversing the blood-brain barrier, which poses a major hurdle to their effective administration. Thus, the primary mode of administration for oligonucleotide-based therapies, especially those targeting the central nervous system, remains either an intraventricular or an intrathecal injection [ 185 ‒ 187]. Additionally, the short half-life of ASOs necessitates prolonged and repeated administration, which inevitably increases treatment costs and impacts patient compliance [188]. While ASOs exhibit a high degree of sequence specificity, off-target effects may lead to unintended gene silencing or activation, which may trigger toxic reactions [186]. Moreover, systematic identification of suitable genetic variants amenable to ASO-based therapy (especially splice-switching therapy) remains challenging. Interestingly, a predictive taxonomy framework was proposed to classify variants based on the predicted severity of splicing disruption and the potential for rescuing mis-splicing via SSO treatment [171]. Variants are categorized as ‘possibly’ or ‘probably’ amenable to splice-switching therapy, providing a systematic approach to expanding the applicability of SSO therapies and accelerating clinical trials for rare genetic disorders [189].

The field of SSO therapies has experienced explosive growth in research as we continue to develop a deeper understanding of RNA splicing mechanisms. The ability of SSOs to directly correct pathogenic splicing events has great potential for next-generation precision medicine approaches [148]. In addition to the well-established roles played by splice site motifs, exon/intron enhancers, and silencers in directing RNA splicing, a growing body of evidence suggests that RNA secondary structure plays a parallel role in guiding this process. This discovery has paved the way for a novel targeting direction for the design of SSO therapies. As a new target for splicing regulation, the RNA secondary structure will surely provide new opportunities for the development of SSO therapy and expand its clinical application prospects in more disease areas.

## Conclusions and Perspectives

Post-transcriptional regulation is a pivotal process for precisely decoding genomic information. The abundant RNA structures within the transcriptome imply a potential structural code that provides additional precision and accuracy for gene expression regulation [190]. In this review, a valuable framework is provided to understand how RNA secondary structures influence alternative splicing. The compromised formation of optimal RNA structures often leads to severe consequences for splicing outcomes and aids in furthering our understanding of the pathological mechanisms underlying severe diseases.

Despite our growing understanding of how the RNA structurome influences splicing, several challenges still remain. First, we scratched the surface of the RNA structurome complex, which is primarily constrained to secondary structures, while the actual folding landscapes within live cells remain largely elusive [14]. A significant future challenge will be to robustly probe RNA structures in living cells and compare the data with *in vitro* refolded RNAs to uncover the unique conformations of cellular RNAs. Undeniably, recent advancements in high-throughput structural probing techniques such as SHAPE-MaP [191], RIC-seq [192] and icSHAPE-MaP [193] have enabled us to comprehensively characterize the entire RNA landscape and precisely identify distinct subsets of highly conserved and functional structural regions. This, in turn, has greatly facilitated the development and design of ASOs. Second, the RNA structurome is not a static entity. RNA can dynamically reshape its structure in response to specific cellular cues, thereby fine-tuning and expanding its functions [ 194, 195]. To comprehensively elucidate the dynamic regulatory mechanisms of the RNA structurome, we still need to develop novel chemical probes in combination with high-resolution transcriptomic techniques and strengthen the close collaboration between experimentalists and computational scientists. This will enable us to analyze the regulatory roles of the RNA structurome in greater spatial and temporal detail [196].

Furthermore, RNA structure has emerged as a potential feature to consider when seeking potential therapeutic approaches. Alternative splicing defects are widespread in human diseases [ 148, 197 ], with many mutations within RNA structural elements closely linked to these conditions [ 198 ‒ 200]. Elucidating the mechanisms by which RNA structure regulates aberrant splicing in diseases may open up opportunities for the discovery of new drug targets, which will be a major research focus in the future. Another intriguing question concerns deciphering the personalized RNA structural landscape of individuals, and resolving this problem may also pave the way for the establishment of precision medicine approaches.
